# Conflicts of interest among dermatology textbook authors^☆^

**DOI:** 10.1016/j.ijwd.2019.08.003

**Published:** 2019-08-12

**Authors:** Jorge Roman, David J. Elpern, John G. Zampella

**Affiliations:** a The Ronald O. Perelman Department of Dermatology, New York University, New York, NY, United States; b The Skin Clinic, Williamstown, MA, United States

## Abstract

**Background:**

Conflict of interest as it relates to medical education is a burgeoning topic of concern. Dermatology textbooks are an influential resource for dermatologists. This study evaluates industry payments to authors of major dermatology textbooks.

**Objective:**

The primary objective of this study is to evaluate whether authors of dermatology textbooks had appreciable conflicts of interest in the form of payments from industry.

**Methods:**

This is a retrospective study in which the authors and editors of eight commonly used general dermatology textbooks were entered into the ProPublica Dollars for Docs database to identify industry payments data from 2016.

**Results:**

The total compensation for 381 authors in 2016 was $5,892,221. Zero payments were reported for 39.6% of authors. Of the dermatologists, 50%, 66%, 70%, and 81% received less than $100, $500, $1000, and $5000, respectively. The top 10% of dermatologists who collected payments received $5,267,494, which represented 89% of the total payment amount.

**Limitations:**

The study was limited to eight textbooks. Data are only as accurate as reported to the Centers for Medicare and Medicaid Services. The database does not include information on dermatologists from non-U.S. institutions. Funding for clinical trials and other avenues of support (e.g., lasers, cosmetic instruments, institutional payments) are also not captured in this database.

**Conclusion:**

A minority of authors of influential dermatology textbooks received the lion’s share of payments from industry.

## Introduction

In our multifarious economic and health care environment, relationships between physicians and industry are under increased scrutiny. Financial relationships between physicians and industry are common for all medical specialties, and dermatology is not immune (Hurley et al., 2014, Sams and Freedberg, 2000, Williams et al., 2006).

In recent years, dermatologists’ relationship with industry has increased immensely. The global pharmaceutical market in dermatology is projected to exceed $34 billion per year by 2023 (Prescient & Strategic Intelligence, 2018). The relationship with industry is a complicated subject. Support from industry has been important for the advancement of dermatology and has provided funding support for a range of activities, including clinical trials, educational materials, and travel support for residents and fellows. These funds are integral for the growth and maintenance of the specialty. For example, exhibit revenue from technical exhibits at large meetings helps support registration and educational costs for attendees and provides funding for other non-income-producing activities. The pervasiveness of industry is incontrovertible and spans a gamut ranging from continuing medical education programs to educational grants to advertisements in journals (Sams and Freedberg, 2000).

Unsurprisingly, the relationship with industry allows for potential conflicts of interest (CoI). Although exposure to industry can begin as early as the medical school years, dermatology residency represents a significant period of time during which residents are the target recipients of certain sponsored educational activities or materials. A few articles have been written about CoI in dermatology as well as the types of interaction between dermatologists and industry (Anstey, 2018, Ashack et al., 2015, Feng et al., 2016), but there is a dearth of literature on the impact of industry relationships as it pertains to dermatology education. In one study, resident physicians from hospitals associated with Mount Sinai School of Medicine showed that most respondents found industry funding of education and industry-supported educational materials useful, despite finding bias in lectures (Korenstein et al., 2010).

One potentially influential educational modality is dermatology textbooks. Textbooks are far-reaching because they are often enduring references used throughout years of clinical practice. These educational keystones describe the breadth of how a disease is defined and include recommendations for treatments. Unlike guidelines that govern disclosures of CoI in most scientific journals, it is not currently common practice for authors/editors to disclose their financial CoI in textbooks. Previous studies that examined potential CoI among authors of biomedical textbooks found an appreciable subset of authors who received compensation from medical product companies (Piper et al., 2015, Piper et al., 2018). In one study of pharmacology textbooks, almost one-third of authors of a single textbook had received money from a pharmaceutical company that was undisclosed to readers. Speaker fees accounted for 28.3% of support, followed by consulting and research at 27% and 23.9% respectively. Additionally, men and academic physicians (i.e., MD/PhD) had a greater likelihood of CoI than female authors (Piper et al., 2015).

Herein, we aim to understand whether authors and editors of influential dermatologic resources have appreciable potential financial CoI in the form of payments from industry using a publicly available database, ProPublica Dollars for Docs (Tigas et al., 2016).

## Methods

### Study sample

The textbooks selected for this study are listed on the American Academy of Dermatology (AAD) website as board preparation resources recommended by members of the AAD Resident and Fellows committee under the category of general dermatology textbooks (AAD, 2018). The most recent editions of eight commonly used books were selected and are listed as follows: *Dermatology* (4th edition, 2017), *Andrews’ Diseases of the Skin: Clinical Dermatology* (12th edition, 2015), *Dermatology Secrets Plus* (5th edition, 2015), *Genodermatoses: A Clinical Guide to Genetic Skin Disorders* (2nd edition, 2004), *Comprehensive Dermatologic Drug Therapy* (3rd edition, 2012), *Hurwitz Clinical Pediatric Dermatology: A Textbook of Skin Disorders of Childhood and Adolescence* (5th edition, 2015), *Dermatology: Illustrated Study Guide and Comprehensive Board Review* (2nd edition, 2017), and *Clinical Dermatology: A Manual of Differential Diagnosis* (3rd edition, 2003).

A list of authors and editors was compiled using the Contributors section of each textbook as well as inspection of each individual chapter. Author and editor names were entered into the ProPublica Dollars for Docs database to identify payment data. Data on payments to physicians are required by the Physician Payment Sunshine Act (part of the Affordable Care Act), and reported to the Centers for Medicare and Medicaid Services (CMS). Under the Physician Payments Sunshine Act, these payments are reported in categories including consulting, speaking fees, food, travel, and research. Pharmacists, physician assistants, nurse practitioners, and biomedical scientists are not currently covered by the Sunshine Act (Kirschner et al., 2014). Information on sex was determined using the Find a Dermatologist tool produced by the AAD, which lists the sex of board-certified dermatologists. For those whose information was not available on the Find a Dermatologist tool, an examination of professional information and biographies on individual practice websites was performed.

This study was reviewed and approved by the New York University School of Medicine’s institutional review board.

### Data analysis

Data analysis was completed using Excel, version 16.16.2. Compensation (US$) was expressed as the median because the distribution was skewed. Standard deviation (SD) was used to report variability. Authors whose primary affiliation was outside of the United States (27.9% of all authors) and non-physicians were excluded from the calculations. Authors in the database but without any reported data were assumed to have received zero payments.

## Results

### Author characteristics

In total, 544 authors and editors were identified, of whom 152 without U.S. affiliations were excluded. Additionally, 11 other authors classified as non-physicians were removed from the analysis (9 PhDs, 1 JD, and 1 medical student). Ultimately, 381 authors were included in the final analysis. Of these recipients, 217 (57%) were men and 164 (43%) were women.

The total compensation for 2016 was $5,892,221, and the total number of payments was 9804. The median total industry payment to authors was $96 (interquartile range, $96-$1726; mean [SD]: $15,465 [$54,815]). This was lower than the 2016 median payment amount for all U.S dermatologists (n = 9180) of $411, as well as the 2016 median payment for all physicians across all specialties of $160 (CMS, 2018). The median number of payments per dermatologist was 1 (mean: 25), which also was lower compared with the median number of payments for all U.S. dermatologists and the median number of payments across all specialties (median: 4 and 14, respectively). Of note, 151 authors (39.6%) had zero payments reported. Additionally, 50%, 66%, 70%, and 81% of dermatologists received less than $100, $500, $1000, and $5000, respectively. The top 10% of dermatologists receiving payments (n = 38) received $5,267,494, which represented 89% of the total payment amount (Table 1).

**Table 1:** Author and payment characteristics.

Total no. of authors	544
Non-U.S. affiliation, n	152
Non-clinicians, n	11
Authors included in analysis, n	381
Men, n (%)	217 (57)
Women, n (%)	164 (43)
2016 payment data	
Total compensation for 2016 (US$)	5,892,221
Total number of payments for 2016	9804
2016 mean (standard deviation) total payment (US$)	15,465 (54,815)
2016 mean of payments, n	25
Authors with zero payments, n (%)	151 (39.6)
Authors who received <$100, n (%)	190 (50)
Authors who received <$500, n (%)	251 (66)
Authors who received <$1000, n (%)	267 (70)
Authors who received <$5000, n (%)	308 (81)
Top 10 % receiving payments	38
Total payment (%) of top 10 percent	5,267,494 (89)

**Fig. 1:**
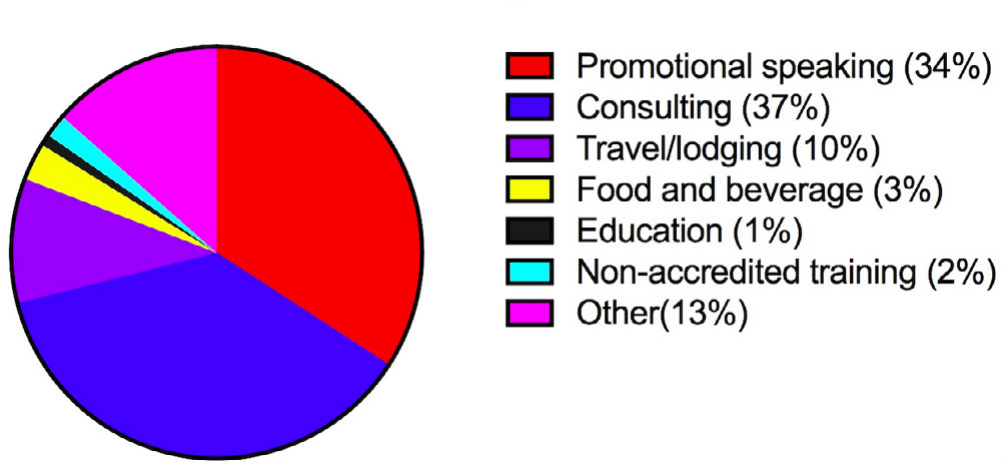
Distribution of payments among authors who received >$10,000.

A separate analysis was performed of authors who received >$10,000 to better characterize the distribution of payment types. The total payment amount for authors in this group was $5,702,476, which represents 96.7% of payments. The median total industry payment for this group was $60,762 (interquartile range, $22,569-$115,965; mean [SD]: $95,041 [$107,492]). The average number of payments per dermatologists in this group was 110. Of these payments (total amount, total percent), speaker fees ($1,947,399; 34.1%), consulting fees ($2,104,449; 36.9%), and travel/lodging payments ($558,547; 9.8%) comprised 80.8% of payments (Fig. 1). Sixty-one percent of recipients in this group were academic dermatologists. The top 15 companies contributing to payments were pharmaceutical manufacturers and paid dermatologists $3,461,765 combined, which represents 60.7% of the total disbursement (Table 2).

**Table 2:** Top payers for 2016.

Top 15 payers to authors earning >$10,000	
Humira (Abbvie)	706,353
Taltz (Eli Lilly)	468,230
Sklice (Sanofi Pasteur)	454,000
Cosentyx (Novartis)	448,137
Xeljanz (Pfizer)	386,509
Stelara (Janssen)	268,232
Otezla (Celgene)	222,292
Enbrel (Amgen)	113,646
Eucrisa (Anacor)	109,233
Ecoza (Exeltis)	101,142
Xolair (Genentech)	71,713
Jublia (Valeant)	39,616
Enstilar (Leo Pharma)	33,561
Remicade (Janssen)	22,271
Simponi (Janssen)	16,830

To evaluate trends of compensation, data were also obtained from the years 2013 to 2015 (Table 3). Total compensation and the number of payments have increased steadily since 2013 for this group. The total compensation and number of payments roughly tripled between 2013 and 2016.

**Table 3:** 2013–2015 payments data.

Year		
2015	Total compensation (USD)	5,323,326
	Total number of payments	9114
	Mean (SD) total payment (USD)	13,971 (45,993)
	Mean number of payments	24
2014	Total compensation (USD)	4,292,310
	Total number of payments	7915
	Mean (SD) total payment (USD)	11,265 (37,023)
	Mean number of payments	21
2013	Total compensation (USD)	1,508,147
	Total number of payments	3070
	Mean (SD) total payment (USD)	3958 (14,648)
	Mean number of payments	8

## Discussion

Over the past several years, legislative measures have been employed to make interactions between physicians and industry more transparent (Agrawal et al., 2013, Kirschner et al., 2014). Unlike in the primary literature, it is not currently common practice for authors to disclose their financial CoI in textbooks. In this study, we characterized payments from industry received by authors of major general dermatology textbooks used as fundamental resources in dermatology resident education and clinical practice. The data from this study showed that the remuneration received by textbook authors was on average less compared with that received by dermatologists at large and compared with physicians across all specialties. Considering that 54% of authors in this study received payments, industry interaction in this cohort was less compared with other groups of dermatologists. In studies by Feng et al., 2016, Checketts et al., 2017, 73.3% and 86% of dermatologists received payments from industry.

However, further analysis showed that payments varied widely across recipients. The payment distribution was skewed with a minority of dermatologists receiving the majority of payments. An analysis of the higher stratum of recipients allowed for characterization of the distribution of payment categories. Compensation for speaking arrangements and consulting fees made up the majority of the total payment amount for this cohort. Travel, lodging payments, and food and beverage payments accounted for a lesser proportion. These findings are comparable with those in other specialties (Campbell et al., 2007, Chang, 2015, Fleischman et al., 2016, Rathi et al., 2015 Samuel et al., 2015, Tierney et al., 2016).

The prevalence of academic dermatologists in this cohort is not surprising given the focus of the study. Sex differences in the amount of money received from industry have been previously reported (Rose et al., 2015). In this cohort, men outnumbered women among top industry payment recipients. The 15 highest-paying manufacturers and most of the companies that made payments to dermatologists in the dataset belong to the pharmaceutical industry (Table 2). The predominance of pharmaceutical payments in dermatology differs from other specialties, such as orthopedic surgery, otolaryngology, and ophthalmology, in which surgical and diagnostic companies provide a greater amount of support (Chang, 2015, Rathi et al., 2015, Samuel et al., 2015).

Given the financial incentives of pharmaceutical companies, the pharmaceutical industry has a particular interest in targeting young physicians in training as they foster their own disease treatment and prescribing patterns. However, our data suggest that only a minority of dermatologists who author textbook chapters have appreciable CoI in the form of industry payments. It stands to reason that both industry companies and textbook makers would select for well-known authorities and leaders in the field. Experts are sought after by industry for the purposes of discussing, testing, or evaluating their products and by textbook publishers to provide their knowledge and expertise. Given that textbooks address the breadth of dermatologic disease, authors of chapters on lesser known or rarer entities are less likely to have ties to industry and as such expected to receive less financial compensation. These authors decrease the mean and median payment amounts of the group at large.

Full disclosure of CoI is essential for readers to reach their own conclusions about the significance of CoI, but several suggestions have been put forth in other articles as strategies to improve transparency. Piper et al. (2018) previously recommended that publishers and editors implement disclosure requirements similar to those present in journal article publications. Piper et al. (2018) also recommended that the disclosure of a contributor’s affiliation and CoI, if any, be placed at the beginning of each chapter rather than within a separate contributors section or other section. The procurement, sharing, and completion of CoI information using a standardized form for all authors and editors, such as the one used by the International Committee on Medical Journal Editors, was also proposed (Drazen, 2010). Other articles have advocated that only individuals without CoI should be eligible to contribute reviews, clinical guidelines, or author educational materials (Cosgrove and Krimsky, 2012, Kearns et al., 2016); however, we believe that financial CoI should not be interpreted in a negative fashion by default in all contexts but rather should be available so that readers can draw their own conclusions. Conversely, other articles contend that disclosure does not have a significant impact or may even adversely affect relationships between different parties (Cain et al., 2011, Pearson et al., 2006). Whether industry payments to authors affect the quality of information in dermatology textbooks for better or for worse remains uncertain.

## Limitations

Generalization of the findings of this study may be limited because only eight general dermatology textbooks were analyzed. Other limitations in this dataset include intrinsic biases related to the collection of data via the Sunshine Act that have been criticized in the past (Babu et al., 2016). Similarly, our study population is limited to physicians; PhDs, nurses, and physician assistants are not included in this study and may represent a fraction of this cohort.

The failure to capture international contributors also is a limitation. Importantly, lasers and other cosmetic instruments are not reimbursed by government-sponsored insurances because companies that specialize in these areas are not required to report to CMS; thus, payments from this category are likely underrepresented in the database.

## Conclusion

The relationships between dermatologists and industry are varied, complex, and robust. This study helps to further characterize the relationship between authors of general dermatology textbooks and industry. Continued discussion to foster transparency among physicians, regulators, and the public with regard to various topics, such as policies, physician behaviors, and the potential for CoI in educational resources, is important.

## Conflict of Interest

None.

## Funding

None.

## Study Approval

The authors confirm that any aspect of the work covered in this manuscript that has involved human patients has been conducted with the ethical approval of all relevant bodies.
